# The Histamine H1 Receptor Participates in the Increased Dorsal Telencephalic Neurogenesis in Embryos from Diabetic Rats

**DOI:** 10.3389/fnins.2017.00676

**Published:** 2017-12-14

**Authors:** Karina H. Solís, Laura I. Méndez, Guadalupe García-López, Néstor F. Díaz, Wendy Portillo, Mónica De Nova-Ocampo, Anayansi Molina-Hernández

**Affiliations:** ^1^Departamento de Fisiología y Desarrollo Celular, Instituto Nacional de Perinatología “Isidro Espinosa de los Reyes”, Mexico City, Mexico; ^2^Programa Institucional de Biomedicina Molecular, Sección de Estudios de Posgrado e Investigación, Insituto Politécnico Nacional, Escuela Nacional de Medicina y Homeopatía, Mexico City, Mexico; ^3^Departamento de Neurobiología Conductual y Cognitiva, Instituto de Neurobiología, Universidad Nacional Autónoma de México, Juriquilla Querétaro, Mexico

**Keywords:** histamine, H_1_ receptor, chlorpheniramine, maternal diabetes, cortical neurogenesis

## Abstract

Increased neuron telencephalic differentiation during deep cortical layer formation has been reported in embryos from diabetic mice. Transitory histaminergic neurons within the mesencephalon/rhombencephalon are responsible for fetal histamine synthesis during development, fibers from this system arrives to the frontal and parietal cortex at embryo day (E) 15. Histamine is a neurogenic factor for cortical neural stem cells *in vitro* through H_1_ receptor (H_1_R) which is highly expressed during corticogenesis in rats and mice. Furthermore, *in utero* administration of an H_1_R antagonist, chlorpheniramine, decreases the neuron markers microtubuline associated protein 2 (MAP2) and forkhead box protein 2. Interestingly, in the diabetic mouse model of diabetes induced with streptozotocin, an increase in fetal neurogenesis in terms of MAP2 expression in the telencephalon is reported at E11.5. Because of the reported effects on cortical neuron differentiation of maternal diabetes in one hand and of histamine in the other, here the participation of histamine and H_1_R on the increased dorsal telencephalic neurogenesis was explored. First, the increased neurogenesis in the dorsal telencephalon at E14 in diabetic rats was corroborated by immunohistochemistry and Western blot. Then, changes during corticogenesis in the level of histamine was analyzed by ELISA and in H_1_R expression by qRT-PCR and Western blot and, finally, we tested H_1_R participation in the increased dorsal telencephalic neurogenesis by the systemic administration of chlorpheniramine. Our results showed a significant increase of histamine at E14 and in the expression of the receptor at E12. The administration of chlorpheniramine to diabetic rats at E12 prevented the increased expression of βIII-tubulin and MAP2 mRNAs (neuron markers) and partially reverted the increased level of MAP2 protein at E14, concluding that H_1_R have an important role in the increased neurogenesis within the dorsal telencephalon of embryos from diabetic rats. This study opens new perspective on the participation of HA and H_1_R receptor in early corticogenesis in health and disease.

## Introduction

Maternal diabetes is a risk factor that increases the incidence of neural tube defects (NTDs) by at least 11/1000 in humans (estimated from: Soler et al., 1976; Copp et al., 2013; Salih et al., 2014; ranging from 1.1 to 4% depending on geographic location). In animal models, such as mice, this percentage increases to 27% (Xu et al., 2013). In addition to NTDs, maternal diabetes has also been linked with impairments in intellectual, verbal coefficient, language, motor activity, learning, and psychosocial development (Ornoy et al., 1998, 1999; Stenninger et al., 1998; Dionne et al., 2008; Nomura et al., 2012; Camprubi Robles et al., 2015). Thus, alternative defects to NTD may arise during central nervous system (CNS) development.

Maternal diabetes may affect neural stem cells (NSC) proliferation, migration, differentiation, and survival. These effects can, in turn, lead to cytoarchitectonic defects that affect neural development and, consequently, to the impairment of diverse CNS functions. The type and extent of these disturbances will depend on which anatomic structure is affected and the time window in which the insult occurs during neural tube development. Indeed, several studies have shown that high glucose levels lead to abnormal NSC death, proliferation, and cell-fate choice both *in vivo* and *in vitro* (Liao et al., 2004; Fu et al., 2006; Chatzigeorgiou et al., 2009; Pavlinkova et al., 2009; Xu et al., 2013).

Fu et al. (2006) reported increases in both neuron and glial differentiation in embryos from diabetic mice within the ventral and dorsal telencephalon at embryo day (E) 11.5 and in NSCs treated with a high glucose concentration *in vitro*. These changes were attributed to the increased expression of the neurogenic factors neurogenin1 (*Ngn1*), *Ngn2*, and Achaete-Scute family basic helix-loop-helix (bHLH) transcription factor 1 (*Mash1*). These authors also reported enhanced expression of Sonic Hedgehog, which may promote neurogenesis through decreased expression of Hes family bHLH transcription factor 1 (*Hes1*) and *Hes5*. In contrast, Xu et al. (2013) reported delayed and decreased neurogenesis during neural tube development (E8) in the same model.

During cortical development in mice, it is estimated that the birth of deep cortical layer neurons begins at E10.5 and ends at E14.5 (Angevine and Sidman, 1961; Gaspard et al., 2008, 2009; Sansom and Livesey, 2009), while in the rat the neurogenesis starts at E12 (deep cortical layer birth; Valverde et al., 1989; Bayer and Altman, 1991).

Additionally, extrinsic and intrinsic factors participate in the chronological programming of NSC proliferation, migration, differentiation, and survival, which lead to the final CNS cytoarchitecture.

In rat, histamine (HA) is one of the first neurotransmitters to be detected in CNS, with the highest concentration at E14 and E16 (Vanhala et al., 1994), coinciding with the peak of neuron differentiation in the cerebral cortex (Gaspard et al., 2008, 2009; Sansom and Livesey, 2009). Fibers from the transient histaminergic neurons in the mesencephalon can be observed through the ventral tegmental area, within the medial forebrain bundle and the optic tract, reaching the frontal and the parietal cortex at E15, earlier than other monoaminergic systems (Specht et al., 1981; Lidov and Molliver, 1982; Auvinen and Panula, 1988; Reiner et al., 1988; Vanhala et al., 1994).

The messenger RNA (mRNA) of the histamine receptors 1 (H_1_R) and 2 (H_2_R) are expressed in the cortical neuroepithelium at E14, while the H_3_R appears in cerebral cortex until E19 (Kinnunen et al., 1998; Heron et al., 2001; Karlstedt et al., 2001, 2003).

HA, as other neurotransmitters, (GABA, serotonin, acetylcholine and glutamate) are important extrinsic factors affecting NSC differentiation *in vitro* (LoTurco et al., 1991; Williams et al., 2004; Zhou et al., 2004; Andang et al., 2008; Andang and Lendahl, 2009). HA acts as a neurogenic extrinsic factor in cortical and mesencephalon NSC through H_1_R activation. In cortical NSC, HA increases the expression of microtubule-associated protein 2 (MAP2), Ngn1, forkhead box protein 2 (FOXP2, a deep cortical layer marker), and Prospero 1 (Prox1), and promotes asymmetric cell division (Molina-Hernandez and Velasco, 2008; Rodriguez-Martinez et al., 2012; Molina-Hernandez et al., 2013). Furthermore, the intrauterine inhibition of the H_1_R at E12 decreases βIII-tubulin (βIII-Tub; a marker for immature neurons) and FOXP2 immunoreactivity in rat cortical neuroepithelium at E14 (Molina-Hernandez et al., 2013).

Given the emerging knowledge on the role of HA in neuron differentiation and the effect of hyperglycemia on neurogenesis, here we investigated whether the levels of HA and/or the expression of the H_1_R increases in embryos from diabetic rats during early corticogenesis and if these play a role in the increased neurogenesis in the dorsal telencephalon at E14.

## Materials and methods

Wistar rats (250–300 g) from (INB-UNAM) were maintained in our animal facilities, house dindividually and maintained in standard conditions (12:12 h light/dark cycle, 21 ± 2°C and 40% relative humidity) with free access to food and water were used. A vaginal smear was performed to confirm the presence of spermatozoids the morning after mating, and this time point was defined as E0.5. All experiments followed both the National Institutes of Health (NIH, USA) “Guide for the Care and Use of Laboratory Animals (NIH Publication No. 80-23, revised 1978)” and the “Norma Official Mexicana para la Producción Cuidado y Uso de Animales de Laboratorio” (NOM-062-ZOO-1999). The accepted protocol number received from the institutional research, biosecurity and ethic committees was 3230-21202-01-2015.

### Diabetes induction and antagonist treatment

At day 5 of pregnancy, pregnant rats received a single intraperitoneal injection of either a buffered citrate solution (vehicle; pH 7.4) for control rats or streptozotocin (STZ; Sigma–Aldrich, St. Louis, MO, USA; body weight: 50 mg/kg) for experimental rats (diabetic rats). From 24 h after vehicle or STZ injection until sacrifice the glucose level was measured daily using a drop of blood taken from the tail vein and a glucometer (ACCU-Chek Performa, Roche Diagnostics, Basel, Switzerland). Rats with glucose levels above 200 mg/dl were included in the diabetic group, while animals with <200 mg/dl levels were discarded.

To explore the possible role of H_1_R in the increased neuron differentiation in embryos from diabetic rats, we performed a series of experiments, where injectable water (vehicle; Laboratorios PiSA S. A. de C. V., GDL. Jal. MEX) or 5 mg/kg of the H_1_R antagonist chlorpheniramine (Sigma–Aldrich; Naranjo and de Naranjo, 1968) was intraperitoneally administrated to control and diabetic pregnant rats, at E12, and evaluated at E14.

### Embryonic tissue processing

To determine the HA and H_1_R levels, we sacrificed pregnant rats by decapitation at E12, E14, E16, E18, and E20. The embryos were extracted, and the total and reabsorbed embryos were registered.

The glucose level was measured immediately after embryo decapitation and then heads were promptly placed in cold Kreb's solution (100 mM NaCl, 2 mM KCl, 0.6 mM KH_2_PO_4_, 12 mM NaHCO_3_, 7 mM glucose, 0.1% phenol red, 0.3% bovine serum albumin, and 3% magnesium sulfate; pH 7.4 and 4°C) for dorsal telencephalon or ventral mesencephalon/rhombencephalon dissection using a stereoscopic microscope (Olympus SZX16, Shinjuku, Tokyo, Japan). Finally, tissue was washed with phosphate-buffered saline (PBS; pH 7.4 and 4°C).

For immunohistochemistry, E14 embryos were fixed by immersion in Bouin's solution (15:15:1 saturated picric aqueous solution:formalin 40%:glacial acetic acid) followed by immersion in 15 and 30% sucrose gradients for 24 h each. After fixation, the embryos were frozen in isopentane (Sigma–Aldrich) and 10 μm coronal slices using a cryostat (Leica CM1850 UV, Wetzlar, Germany) were recovered.

### HA level measurement using enzyme-linked immunosorbent assay (ELISA)

We used the ELISA technique (sensitivity: 0.2 ng/ml; 100% HA specificity) to determine the HA level following the protocol recommended by the supplier (ALPCO® immunoassays, Salem, NH, USA). Tissues from the dorsal telencephalon (E12–E20), placenta (E14) and serum of pregnant rat (E14; 50 μl) were used.

The embryo or placenta tissue was homogenized in 100 μl of cold PBS (pH 7.4; POLYTRON PT 2100 Homogenizer, Kinematica, Switzerland) and centrifuged at 10,500 × g for 5 min. Then 50 μl of the supernatant (or serum) was used to determine the HA concentration.

The absorbance values were measured at 495 nm in a multiple detection system (GLOMAX, Promega, Madison, WI, USA). The HA concentration was determined using a reference curve constructed using 0, 0.5, 1.5, 5, 15, and 50 ng/ml HA, and the results were corrected by the amount of protein per sample and expressed as molarity. The protein concentration was determined using the Bradford method (Bradford, 1976).

### Immunohistochemistry

Immunofluorescence procedures were conducted using standard protocols (Molina-Hernandez and Velasco, 2008) in frontal telencephalic coronal slices from fixed embryos at E14. After blocking and permeating (10% normal goat serum and 0.3% Triton-X100 in PBS), the tissues were incubated at 4°C overnight with mouse monoclonal MAP2 (RRID:AB_369978, 1:500; GeneTex, Simpson, PA, USA) and βIII-Tub (RRID:AB_385733, 1:1000; GeneTex) primary antibodies, followed by fluorescent secondary antibodies Alexa-Fluor 568 goat anti-mouse IgG (RRID:AB_2534072, 1:1,000; Thermo Fisher Scientific, Waltham, MA, USA) and Alexa-Fluor 488 goat anti-rabbit (RRID:AB_143165, 1:1,000; Thermo Fisher Scientific) for 1 h at room temperature. Primary antibody incubation was omitted to create a negative control. Nuclei were stained with 4′,6-diamidino-2-phenylindole (DAPI, 1 ng/ml, Sigma), and an epifluorescence microscope (Olympus IX81, Japan) with a charge-coupled device (CCD) camera (Hamamatsu, ORCA-Flash 2.8, Hamamatsu, Japan) was used to obtain images, which were then processed using Adobe Photoshop CS6 (San Jose, CA, USA).

### Quantitative reverse-transcription polymerase chain reaction (qRT-PCR)

qRT-PCR was performed to analyze the temporal expression of H_1_R and histidine decarboxylase (HDC; EC 4.1.1.22, enzyme responsible for convert histidine to histamine) as the relative expression to E12 and differences between groups in terms of the relative expression to the control of H_1_R, HDC, neurogenic factors (Prox1 and Ngn1), and neuron markers (βIII-Tub and MAP2). The dorsal cortical neuroepithelium (for H_1_R, Prox1, Ngn1, βIII-Tub, and Map2) or mesencephalon/rhombencephalon neuroepithelia (for HDC) were obtained and immediately stored at −80°C until use.

Total RNA from E12-E14 (4 epithelia per experiment) and E16-E18 (2 epithelia per experiment) cortical neuroepithelia were isolated using TRIZOL reagent (Invitrogen). The RNA integrity was determined by visualizing 18S and 28S ribosomal RNA stained with ethidium bromide (0.2 mg/ml) in 2% agarose gel. One microgram of RNA was used for the retro-transcription reaction with 0.5 μg of oligo-dT, 1 mM dNTPs, 0.2 mM dithiothreitol (DTT), 1U of RNase inhibitor, and 15U of Super Script® III (Invitrogen). The reaction was incubated at 25°C for 5 min and then at 50°C for 1 h, and the reaction was stopped at 70°C for 15 min. Dynamic ranges were measured for each gene before the qPCR analysis to determine the fluorescence threshold and reaction efficiency.

PCR analysis was performed using 800 ng (H_1_R) or 400 ng (other mRNAs) of cDNA, 20 pmol of forward (F) and reverse (R) primers, and the commercial KAPA™ SYBER FAST® qPCR mix (KAPA Biosystems, Wilmington, MA, USA) with a Rotor-Gene 6000 thermocycler (Qiagen, Germantown, MD, USA). The amplification conditions were as follows: 95°C for 10 min, followed by 35 cycles at 56°C (H_1_R and glyceraldehyde 3-phosphate dehydrogenase [GAPDH]) or 62°C (Prox1, Ngn1, βIII-Tub, and Map2) for 15 s, and a final amplification at 72°C for 30 s. The relative fold changes (relative expression) were determined using the mathematical algorithm 2^−ΔΔCT^, where CT is the cycle threshold (Livak and Schmittgen, 2001). GAPDH was used as the internal control to obtain ΔCT values per sample. After PCR was performed, denaturation curves were calculated to confirm the amplification of a single product (Kubista et al., 2006).

The primer sequences and product size were as follows: H_1_R (292bp), F:5′-CTTCTACCTCCCCACTTTGCT-3′ and R: 5′-TTCCCTTTCCCCCTCTTG-3′; Prox1 (384 bp), F: 5′-TGTTCTTTTACACCCGTTACCC-3′ and R: 5′-CACTATCCAGCTTGCAGATGAC-3′; Ngn1 (131 bp), F: 5′-AGCCCGGCCAGCGATACAGA-3′ and R: 5′-GGACCACCCGGGCCATAGGT-3′; βIII-Tub (102bp), F: 5′-GCCAAGTTCTGGGAGGCTCATC-3′ and R: 5′-GTAGTAGACACTGTAGCGTTCCA-3′; MAP2 (132 bp), F: 5′-GAG AAG GAG GCC CAA CAC AA-3′ and R: 5′-TCTTCGAGGCTTCTTCCAGTG-3′; and GAPDH (189 bp), F: 5′-GGA CCT CAT GGC CTA CAT GG-3′ and R: 5′-CCCCTCCTGTTGTTATGGGG-3′.

Adult rat cerebral cortex or U373MG (CVCL_2219) cell cDNA was used as the positive control, and U373MG was transfected with a rat Hrh1 siRNA-Smart-pool siRNA (Accell, GE Healthcare, Chicago, IL, USA) as a negative control for H_1_R amplification. Endpoint PCR and electrophoresis in 2% agarose gel were performed to verify the size of the products with GelRed (Biotium, Inc., Fremont, CA, USA). Finally, fragments were purified for sequencing.

### Western blot analysis

Protein extracts from a pool of dorsal telencephalon (E12-E14, whole litter and E16-E20, 4 embryos) or ventral mesencephalon/rhombencephalon (E12–E16, whole litter) were obtained after lysis in buffer containing 2.5 mM Tris-HCl pH7.5, IGEPAL 1%, 100 mM NaCl, and protease inhibitors (AMRESCO, Solon, OH, USA). Total protein was determined by the Bradford method (Bradford, 1976).

Denaturalizing protein electrophoresis in 10 or 8% (for MAP2) acrylamide gel was performed with 80 μg (H_1_R and MAP2) or 40 μg (HDC and βIII-Tub) of total protein using a MiniProtean II system (Bio-Rad, Hercules, CA, USA). Proteins were transferred to nitrocellulose membranes (Amersham TM Hybond TM-ECL, Buckinghamshire, UK) using the Trans-Blot® semi-dry transfer cell system (Bio-Rad), as described previously (Villanueva, 2008). The primary antibodies were as follows: rabbit polyclonal anti-H_1_R (RRID:AB_2277328, 1:2,000; Santa Cruz Biotechnology, Dallas, TX, USA), mouse monoclonal anti-MAP2 (RRID:AB_369978, 1:5,000; GeneTex), rabbit monoclonal anti-βIII-Tub (RRID:AB_10620409, 1:2,000; GeneTex), rabbit polyclonal anti-HDC (RRID:AB_2248239, 1:1,500; Santa Cruz Biotechnology), rabbit polyclonal anti-GAPDH (RRID:AB_10167668, 1:1,500; Santa Cruz Biotechnology), mouse monoclonal anti-GAPDH (RRID:AB_11174761, 1:20,000; GeneTex), and anti-actin (RRID:AB_630833, 1:5,000; Santa Cruz Biotechnology). Secondary horseradish peroxidase-conjugated antibodies (RRID:AB_631736 and RRID:AB_631746, 1:15,000; Santa Cruz Biotechnology) or infrared 700nm (RRID:AB_10953628) and 800 nm (RRID:AB_621848) LI-COR secondary antibodies were used (LI-COR, Lincoln, NE, USA). For horseradish peroxidase-conjugated antibodies, luminol (Santa Cruz Biotechnology) was employed to visualize the bands in autoradiographic film (Amersham HyperfilmTM ECL). The blots were digitalized and analyzed by densitometry using ImageJ (http://imagej.nih.gov/ij/, NIH, USA). When infrared secondary antibodies were used, fluorescence intensity analysis was performed with the Odyssey CLx system and Image Studio ver.4.0 (LI-COR).

GAPDH or actin were used as the internal controls. Because a non-specific band was observed in the H_1_R immunoblots and to ensure H_1_R detection, a cell line that highly expresses H_1_R, the U373MG glioblastoma cells were transfected with H_1_R siRNA-Smart-pool or scramble sequences (Accell, GE Healthcare) were used as negative or positive controls, respectively (**Figure 5B**).

### Enzymatic activity

A modified method was used to measure the HDC activity (Keeling et al., 1984; Barnes and Hough, 2002; Shoji et al., 2006). Five hundred milligrams of mesencephalon/rhombencephalon tissue (where fetal histaminergic transitory neurons reside) was obtained from E14 and E16, or from placenta at E14. The tissues were homogenized in 2.5 ml of cold PBS (0.02 M, pH 6.2) containing 20 μM pyridoxal phosphate, 200 μM DTT, and 25 mg phosphorylated cellulose (pH 7.5). The homogenate was then centrifuged at 10,000 × g at 4°C for 15 min, and the supernatant was diluted 1:1 with the enzyme reaction solution (0.1 M PBS pH 6.7, 50 mM pyridoxal phosphate, 100 mM aminoguanidine, 2 mM of L-histidine as a substrate, and 30 μM of histamine N-methyltransferase inhibitor SKF 91488). The mixture was incubated at 37°C for 5 min, and the reaction was terminated by the addition of 0.4 ml of perchloric acid (2.8 M).

The synthesized HA was separated by ion-exchange chromatography using a phosphorylated cellulose support. HA was eluted with perchloric acid (2.8 M) and quantified by ELISA.

### Experimental design and statistical analysis

Sample size was estimated for independent samples and studies analyzed by *t*-test with the power and sample size program (Casagrande et al., 1978; Dupont and Plummer, 1990) using the following parameters: power = 0.9, α = 0.05, δ = 1, σ = 0.7 and *m* = 3. A minimum of four experiments per group were included.

Unpaired *t*-test was performed for comparison between control and diabetic. For temporal analysis and comparisons between control, chlorfeniramine, diabetic, and diabetic+chlorpheniramine groups ANOVA followed the uncorrected Fisher's LSD multiple comparison test were performed. Statistical analysis and graphic creation were performed in GraphPad Prism version 6.0 (GraphPad Software, Inc., La Jolla, CA, USA).

## Results

The glucose levels in control embryos were lower than those in pregnant rats, and “normal” glycemic was not reached until E18, in contrast, diabetic embryos showed significant increased blood glucose levels in all days evaluated with respect diabetic pregnant rats. As expected, glycemic changes in embryos from the diabetic group presented significantly higher glucose levels than the control group embryos during all stages of development, exhibiting values >200 mg/dl starting at E14 (Table 1).

**Table 1:** Glycemic values in control and diabetic pregnant rats and its corresponding litter.

**Post-induction day**	**Glycemia in pregnant rat mean ± S.E.M. (mg/dl) (n)**		**Embryo Day**	**Glycemia in embryo mean ± S.E.M. (mg/dl) (*n* = litter)**	
	**Control**	**Diabetic**		**Control**	**Diabetic**
0	96.28 ± 1.06 (21)	91.3 ± 1.16 (20)			
2	96.58 ± 2.14 (12)	363.23 ± 27.66 (17)			
4	96.41 ± 3.16 (12)	367.92 ± 21.16 (13)			
5	94.30 ± 1.88 (10)	350 ± 30.53 (9)			
6	90.70 ± 2.16 (10)	470.28 ± 24.95 (7)			
7	100 ± 1.08 (12)	414.12 ± 12.56 (25)	12	44.43 ± 0.7^***^ (5)	65.56 ± 4.73^***^; ^*b*^ (5)
9	91 ± 0.77 (12)	501.7 ± 11.24 (10)	14	51.08 ± 3.85^***^ (5)	366.6 ± 20.98^***^; ^*a*^ (5)
11	92 ± 4.9 (8)	542.77 ± 9 (18)	16	63.23 ± 3.04^***^ (5)	310.32 ± 6.35^***^; ^*a*^ (5)
13	91.2 ± 1.25 (5)	395 ± 1.62 (8)	18	89.6 ± 0.7 (4)	453.9 ± 17.95^***^; ^*a*^ (4)
15	90.66 ± 4.63 (3)	550.83 ± 13.15 (10)	20	91.11 ± 2 (3)	432.25 ± 7.81^***^; ^*a*^ (4)

*Differences in the glucose levels between pregnant rats and its litters, and between embryos from control and diabetic pregnant rats were evaluated*.

*** *p < 0.001 vs. maternal*.

a p < 0.001 and

b *p < 0.01 vs. Ctl embryo*.

### Embryos from diabetic rats presented increased neuron differentiation

**Figure 1:**
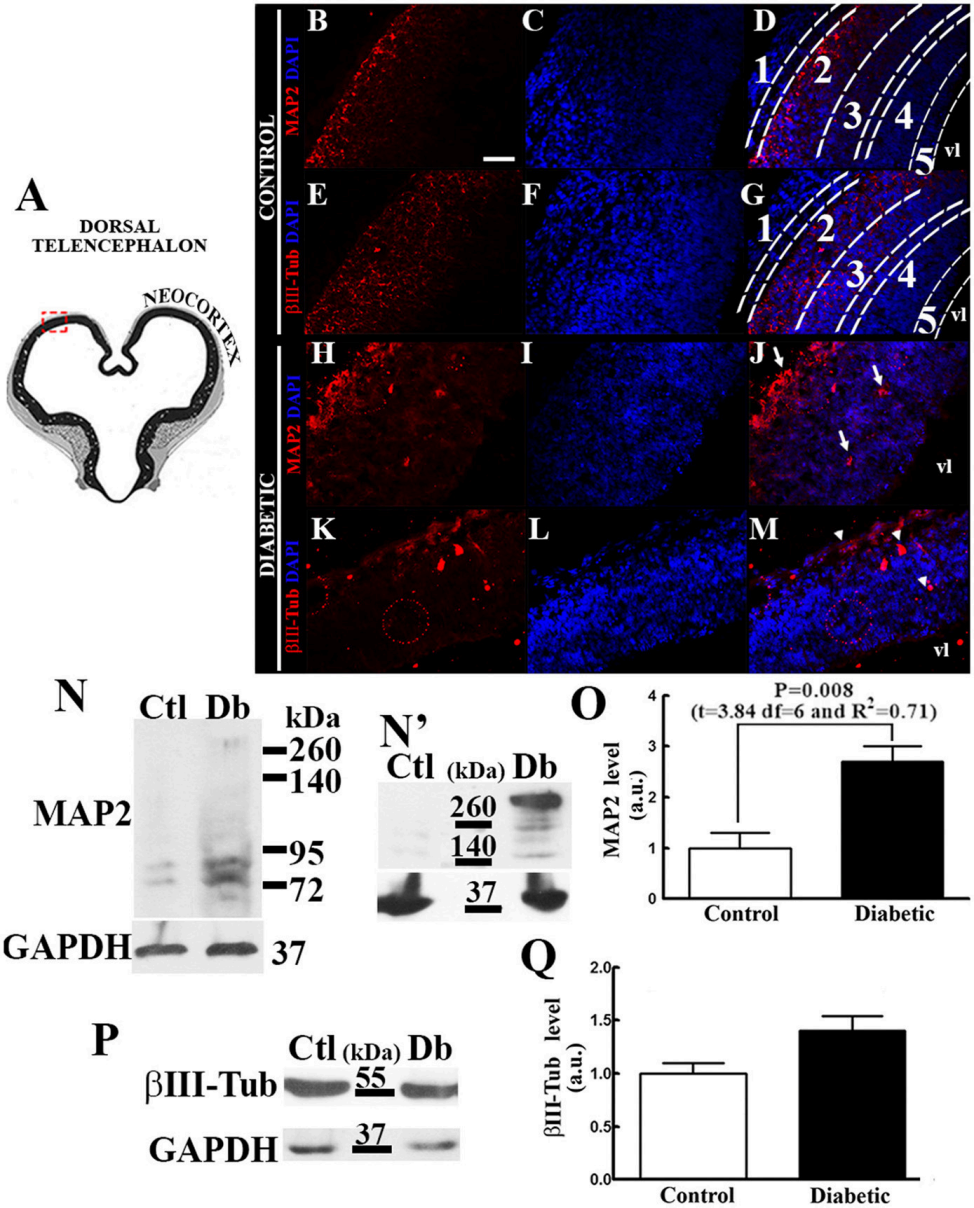
Diabetes during pregnancy increases neuron differentiation and maturation. **(A)** Coronal view of the telencephalon at E14 (modified from Altman and Bayer, 1995). The area outlined by the red dashed line corresponds to the area shown by the micrographs in images **(B–M)**. **(B–M)** Representative epifluorescence micrographs (40×) of immunohistochemistry from the control **(B–G)** and diabetic **(H–M)** groups for MAP2 and βIII-Tub (both in red). Nuclei were stained with DAPI (blue). 1, marginal zone; 2, cortical plate; 3, subplate; 4, subventricular zone; 5, ventricular zone; and vl, ventricular lumen. Arrows in **(J)** and arrows heads in **(M)** indicate signal out from the cortical plate. Scale bar in B = 50 μm. **(N,P)**, representative Western blots for MAP2 **(N)**, βIII-Tub **(P)**, GAPDH (**N,P**; internal control) and an overexposed film from the control (Ctl) and diabetic (Db) groups of the high weight MAP2 band (up in **N'**; >260 kDa) and GAPDH (down in **N'** internal control). **(O,Q)** graphs obtained from the densitometry analysis of MAP2 (lower band ~70 kDa) and βIII-Tub (56 kDa), respectively. Data are the mean (standard error of the mean; S.E.M.) of the optical density expressed in arbitrary units (a.u.) from four independent experiments. Significant *P*-value after unpaired *t-*test are shown in the graph.

Increased dorsal telencephalic neurogenesis in diabetic rat embryos was evidenced by immunohistochemistry and Western blot analyses. In control embryos, MAP2 immunocytochemistry was localized in the cortical plate, and βIII-Tub was observed in the cortical plate and subplate (Figures 1A–G). In addition to a disorganized epithelium making it difficult to distinguish the layers at the time of development evaluated, the distributions of both neuron markers were altered in the diabetic group: MAP2 was observed throughout the neuroepithelium showing an intense mark in what is suggested to be the marginal zone and cortical plate, whereas βIII-Tub exhibited an apical distribution in which its suggested to be the marginal zone (Figures 1H–M). Western blot analysis revealed that embryos from diabetic rats presented significantly increased MAP2c (70 kDa; Figures 1N,O). A high-molecular weight band corresponding to MAP2a/b (>260 kDa; Figure 1N) not observed in control embryos was barely observed in the diabetic. After overexposing the autoradiography film in the area which the high-molecular weight band was observed, this was clearly observed in the diabetic group (Figure 1N'). No significant changes were obtained for βIII-Tub (Figures 1P,Q).

### Increased HA level in the embryonic dorsal telencephalon from diabetic rats at E14

To explore the roles of HA and H_1_R in increased neuron differentiation, we first determined the levels of HA and H_1_R in the cerebral cortex neuroepithelium of embryos from both diabetic and control rats.

**Figure 2:**
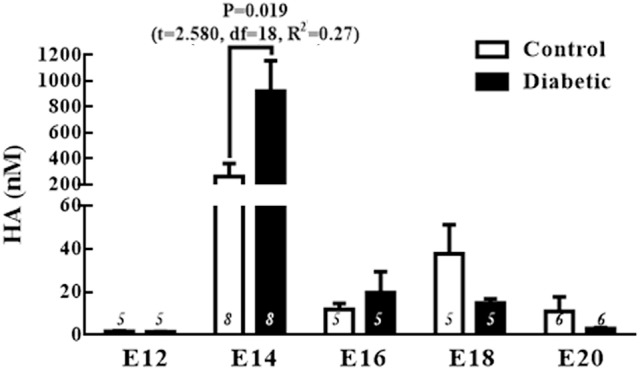
Fetal histamine levels during corticogenesis. The graph shows the histamine (HA) concentrations (nM) in the control and diabetic groups in telencephalic (E12-E20) tissue. Data are the mean (S.E.M.) from 5 to 8 experiments per triplicate (*n* is shown in bars). For changes between different embryo days within the same group, one-way ANOVA was performed [Control, *F*_(4, 27)_ = 3.1, *P* = 0.03, and *R*^2^ = 0.3; Diabetic, *F*_(4, 27)_ = 3.1, *P* = 0.03, and *R*^2^ = 0.3] followed by Fisher's LSD test, *P* = 0.01 in the control for E14 vs. E12 and DF = 26, and in the diabetic *P* = 0.01 for E14 vs. E12 and DF = 26. For comparison between groups, unpaired *t-*test was used and significant *P-*value is shown in the graph. E = embryo day.

The temporal patterns of the HA levels during corticogenesis revealed a significant increase in the HA concentration at E14 in both groups. Comparing the groups revealed that HA level in the diabetic group was 3.5 times higher at E14 than in the control group (Figure 2).

Since we presume that the main source of fetal HA was the transient histaminergic neurons (Vanhala et al., 1994), we measured HDC mRNA, its protein levels (at E12, E14, and E16) and activity (E14 and E16) in ventral mesencephalon/rhombencephalon fetal tissue where fetal HDC expression and HA positive neurons reside.

**Figure 3:**
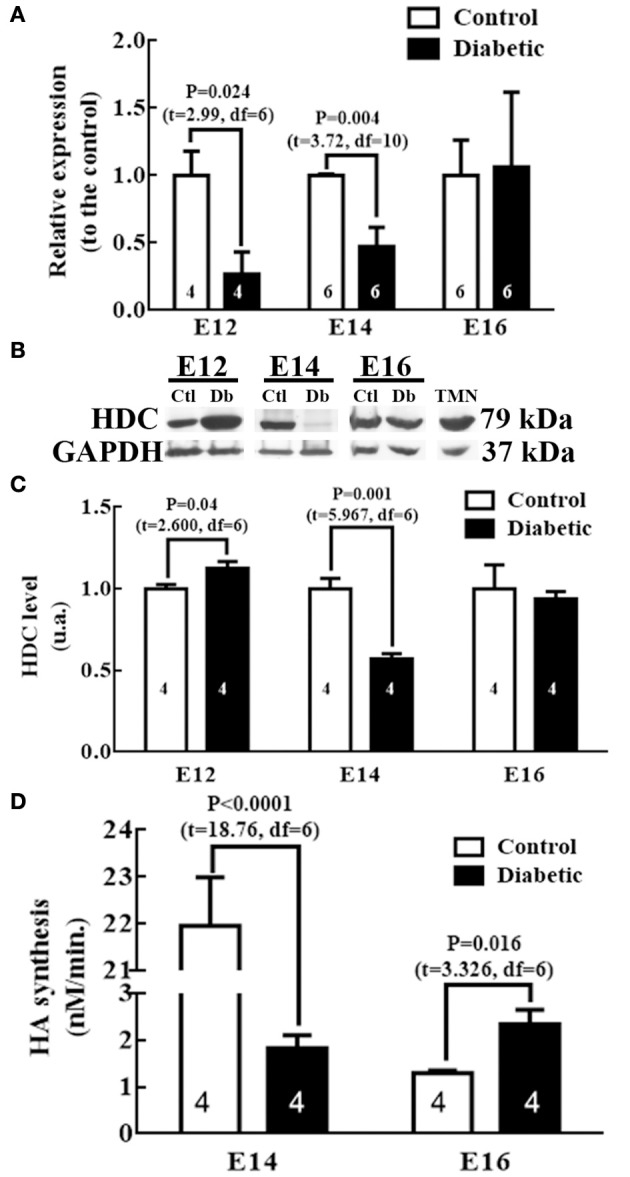
HDC expression and activity in mesencephalic/rhombencephalic tissue. **(A)** Graph of HDC in control and diabetic groups. The results are the relative expression to the control (S.E.M.) using the 2^−ΔΔCT^ method from 4 to 6 experiments per triplicate (*n* is shown within bars). **(B)** Representative Western blots for HDC and GAPDH (internal control) from four experiments in control (Ctl), diabetic (Db) and adult tuberomammillary nucleus tissue (TMN; positive control). **(C)** Graphs obtained from the densitometry analysis of HDC; the results are expressed as the mean (S.E.M.) of the optical density in arbitrary units (a.u.) from four independent experiments. **(D)** Graph representing the HDC activity expressed in nM of HA synthesized per minute; the results are the mean (S.E.M.) from four independent experiments per triplicate. In all graphs, unpaired *t*-test was performed, and significant *P*-values are shown in the graph. E = embryo day.

The results showed significantly lower levels of HDC mRNA in embryos from diabetic rats at E12 (3.8 times) and E14 (2.1 times; Figure 3A). The decreased expression of HDC observed in embryos from the diabetic group was consistent with a decrease in the level of its protein at E14 (1.8 times), however, a significant increase was obtained at E12 with respect the control group (Figures 3B,C). No amplification was detected for HDC at E14 in dorsal telencephalon tissue (data not shown).

Our results revealed a significant decrease at E14 and an increase at E16 in the diabetic group embryos relative to the control embryos (Figure 3D). As a result of the amount of tissue required to assess the HDC activity, this was not determined at E12.

**Figure 4:**
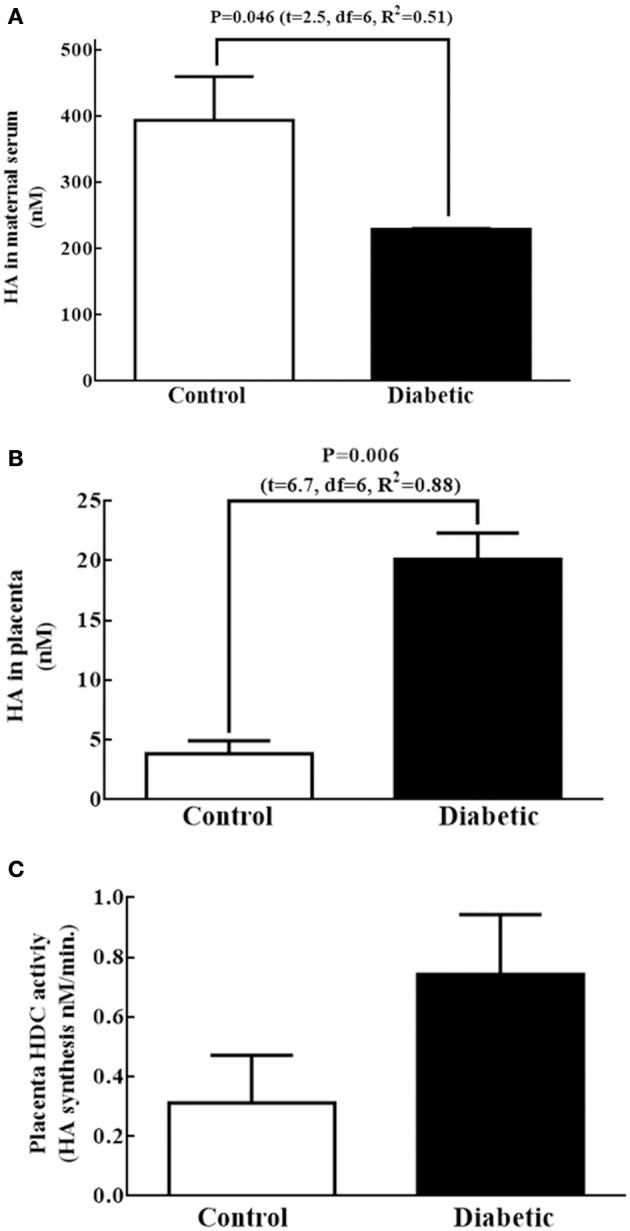
Other possible fetal sources of histamine at E14. **(A,B)** Graphs of the histamine (HA) concentration (nM) in maternal serum and placenta, respectively; bars correspond to the mean (S.E.M.) from four experiments per triplicate. **(C)** Graph representing the HDC activity expressed in nM of HA synthesized per minute in placenta. Bars correspond to the mean (S.E.M.) from four assays per triplicate. Unpaired *t*-test was performed, and significant *P*-values are shown in the graph.

Because the increased level of telencephalon HA at E14 in the diabetic group was not explained by the fetal HDC mRNA, protein level or the HDC activity, other sources, such as the mother or placenta, likely provide HA to the embryos. The HA concentrations in the diabetic maternal serum was lower with respect to the control (Figure 4A). When the levels of HA and HDC activity were evaluated in the placenta, we found that the HA concentration in the diabetic group was 2.3 times higher than in control placentas (Figure 4B). Although there was a tendency to increase, no significant difference in the HDC activity was observed between groups in placenta HDC activity (Figure 4C).

### Altered expression of H_1_R during corticogenesis in embryos from diabetic rats

A nucleotide BLAST for the sequence obtained from the H_1_R PCR product showed 100% identity (nucleotides 594–885 from NM_017018.1; https://blast.ncbi.nlm.nih.gov).

**Figure 5:**
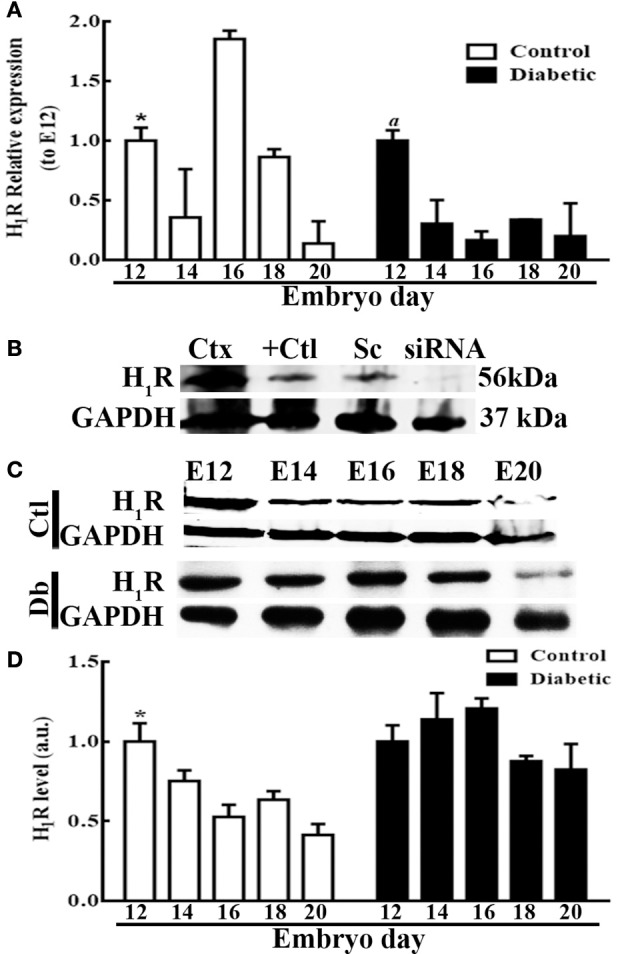
Temporal fetal H_1_R expression during corticogenesis in embryos from control and diabetic rats. **(A)** Graph of the temporal expression analysis of H_1_R in dorsal telencephalic (E12–E20) tissue from control (white bars) and diabetic groups (black bars). Data are the relative expression to E12 (S.E.M.) using the 2^−ΔΔCT^ method from eight experiments per triplicate. One-way ANOVA was performed (Control, *F*_(4, 31)_ = 8.6, *P* < 0.0001 and *R*^2^ = 0.5; Diabetic, *F*_(4, 31)_ = 7.2, *P* = 0.0003 and *R*^2^ = 0.5), followed by Fisher's LDSs test, significant *P*-values in the control for ^*^E12 vs. E14, E16, and E20 were: *P* = 0.03 (*t* = 2.2), *P* = 0.007 (*t* = 2.9), and *P* = 0.02 (*t* = 2.4) respectively, and DF = 31. And in the diabetic for ^*a*^E12 vs. E14, E16, E18, and E20 were: *P* = 0.0004 (*t* = 3.9), *P* < 0.0001 (*t* = 4.7), *P* = 0.0007 (*t* = 3.8), and *P* = 0.0008 (*t* = 3.7) respectively, and DF = 31. **(B)** Western blot from three positive controls (Ctx = adult rat cerebral cortex, U373MG = glioblastoma cell line, and Sc = U373MG cells lipofected with scramble siRNAs) and one negative control (siRNA = U373MG cells lipofected with a pool of H1R-siRNA) for H_1_R (56 KDa) and GAPDH (37 KDa; internal control). **(C)** Representative temporal Western blots for H_1_R and GAPDH from control and diabetic samples. **(D)** Temporal densitometry analysis of H_1_R per group. The results are expressed as the mean (S.E.M.) of the normalized optical density for the control (white bars) and diabetic groups (black bars) expressed in arbitrary units (a.u.) from four independent experiments. One-way ANOVA was performed (Control, *F*_(4, 15)_ = 8.2, *P* = 0.001, and *R*^2^ = 0.7; Diabetic, *F*_(4, 15)_ = 2, *P* = 0.15, and *R*^2^ = 0.34), followed by Fisher's LDS test, significant P values in the control for ^*^E12 vs. E14, E16, E18, and E20 were: *P* = 0.04 (*t* = 2.2), *P* = 0.0007 (*t* = 4.3), *P* = 0.005 (*t* = 3.3), and *P* < 0.0001 (*t* = 5.3) respectively, and DF = 15. E = embryonic day.

The H_1_R mRNA temporal analysis revealed significant changes relative to E12, with the lowest levels at E14 (2.7 times) and E20 (7.7 times) and the highest at E16 (1.9 times). In contrast, in the diabetic group, the highest expression of the receptor was obtained at E12, and this value was statistically different from those recorded for embryos at other ages (Figure 5A). The H_1_R protein levels were significantly decreased at E14 (1.4 times), E16 (1.9 times), E18 (1.6 times), and E20 (2.4 times) compared with E12 in the control group. Interestingly, no temporal changes in the receptor protein levels were observed in the diabetic group (Figures 5C,D).

**Figure 6:**
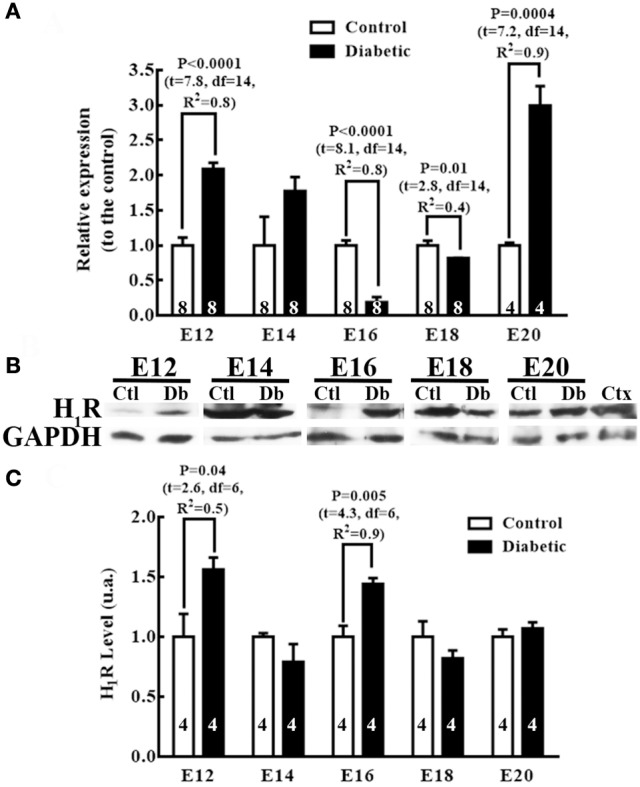
Differences in histamine type 1 receptor expression between embryos from control and diabetic rats. **(A)** Expression of H_1_R in the dorsal telencephalic (E12–E20) tissue from control and diabetic rats. Data are the relative expression to the control (S.E.M.) using the 2^−ΔΔCT^ method from 4 to 8 experiments per triplicate (n is shown within bars). Unpaired *t*-test was performed, and significant P values are shown in the graph. **(B)** Representative Western blots for H_1_R (56 kDa) and GAPDH (37 kDa; internal control) per embryo day (E) in control (Ctl) and diabetic (Db) groups; adult cerebral cortex (Ctx) was used as the positive control. **(C)** Graph of the densitometry analysis of H_1_R; data are shown as the mean (S.E.M.) of the protein level expressed as arbitrary units (a.u.) from four experiments. Unpaired *t*-test was performed, and significant P values are shown in the graph.

Comparing the groups' H_1_R mRNA levels showed that embryos from diabetic rats presented significant increases at E12 (2 times) and E20 (2.9 times), and significantly lower levels at E16 (5.5 times) and E18 (1.3 times) relative to the control group (Figure 6A). However, in the telencephalic tissue from diabetic dams, the protein levels increased significantly at E12 (1.6 times) and E16 (1.7 times) relative to the control group (Figures 6B,C).

### Systemic administration of an H_1_R antagonist prevents increased neuronal markers at E14 in diabetic embryos

BLAST for the sequences obtained from the PCR products showed 100% identity for Prox1 (nucleotides 2248–2631 from NM_001107201), Ngn1 (nucleotides 780–910 from NM_019207.1), βIII-Tub (nucleotides 101–202 from NM_139254), and MAP2 (nucleotides 433–564 from NM_013066.1; https://blast.ncbi.nlm.nih.gov).

**Figure 7:**
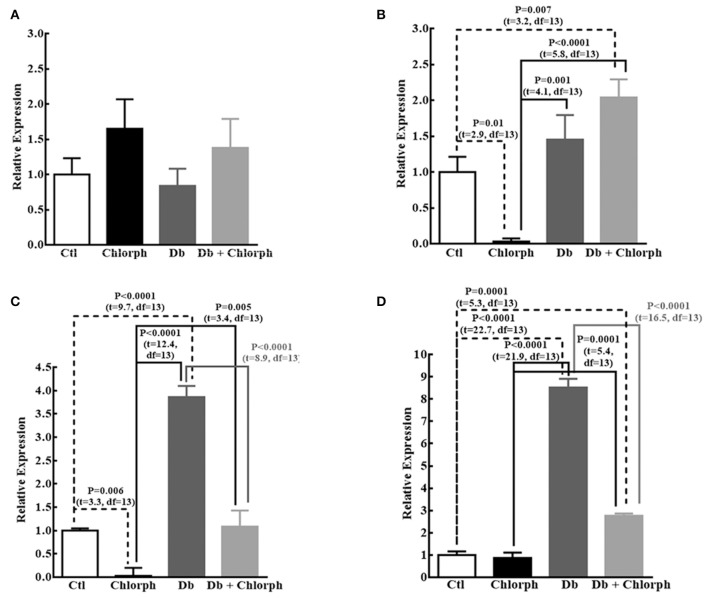
Chlorpheniramine prevents the increased expression of neuronal factors at E14. **(A–D)** Graphs of the expression of Prox1 **(A)**, Ngn1 **(B)**, βIII-Tub **(C)**, and MAP2 **(D)** in telencephalic tissue at E14 from control (Ctl), chlorpheniramine (Chlorph), diabetic (Db) and Db+Chlorph groups. The results are the relative expression to the control (S.E.M.) using the 2^−ΔΔCT^ method from five (Ctl) or four (Chlorph, Dd and Db+Chlorph) independent experiments per triplicate. One-way ANOVA was performed [**A**, *F*_(3, 13)_ = 1.2, *P* = 0.34, and *R*^2^ = 0.22; **B**, *F*_(3, 13)_ = 12.2, *P* = 0.0004, and *R*^2^ = 0.73; **C**, *F*_(3, 13)_ = 57, *P* < 0.0001, and *R*^2^ = 0.93 and **D**, *F*_(3, 13)_ = 218.2, *P* < 0.0001, and *R*^2^ = 0.98], followed by Fisher's LDS multiple comparison test, and significant P values are shown in each graph.

We found a tendency to rise in the expression of Ngn1 (1.5 times; *P* = 0.089) and significant increases in βIII-Tub (3.8 times), and MAP2 (8.2 times) mRNAs in diabetic embryos compared to the control group. Chlorpheniramine administration at E12 prevented the increases of βIII-Tub and MAP2 in the diabetic group and notably reduced the expression of Ngn1 (31.3 less) and βIII-Tub (34.5 less; Figure 7). No significant changes were obtained for Prox1 (Figure 7A).

**Figure 8:**
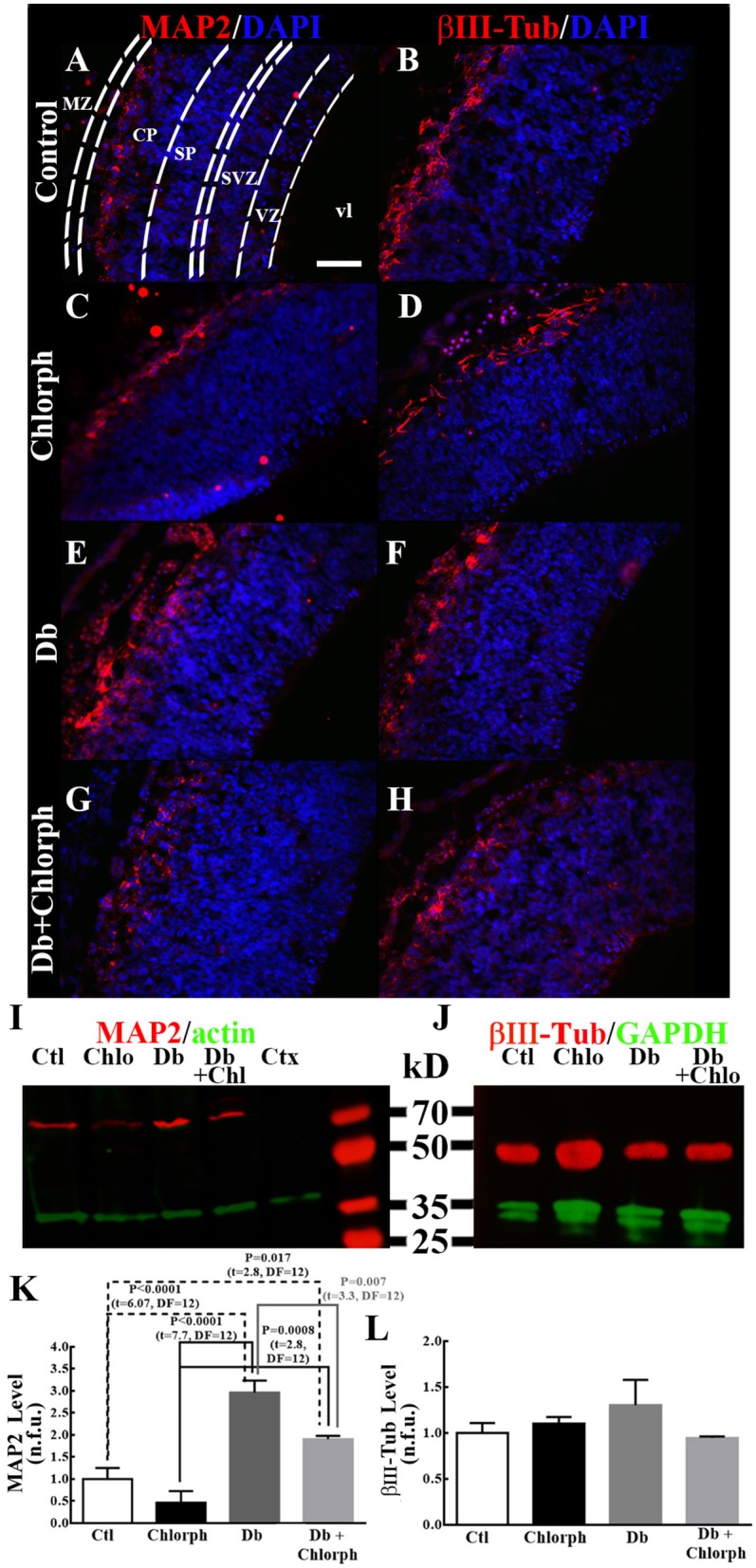
Chlorpheniramine partially prevents increased MAP2 in the dorsal telencephalons of embryos from diabetic rats. **(A–I)** Representative micrographs (40×) of the immunofluorescence of MAP2 and βIII-Tub in control (Ctl; **A,B**), chlorpheniramine (Chlorph; **C,D**), diabetic (Db; **E,F**) and diabetic + chlorpheniramine (Db+Chlorph; **G,H**) groups. MZ = marginal zone, CP = cortical plate, SVZ = subventricular zone, VZ = ventricular zone and vl = ventricular lumen. Scale bar = 50 μm. **(I,J)** Representative Western blots for MAP2 (~70 kDa), βIII-Tub (56 kDa) and as internal controls actin (42 kDa) or GAPDH (37 kDa). Ctl = Control, Chlo = Chloropheniramine, Db = Diabetic, and Db+Chlo = Diabetic+Chloropheniramine. **(K,L)** Graphs of the fluorometric analyses of MAP2 and βIII-Tub, respectively. Data are the mean (S.E.M.) of the fluorescence expressed in normalized florescent units (n.f.u.) from four independent experiments. One-way ANOVA was performed [**K**, *F*_(3, 12)_ = 22.9, *P* < 0.0001, and *R*^2^ = 0.85, and **L**, *F*_(3, 12)_ = 1.06, *P* = 0.4 and *R*^2^ = 0.21], followed by Fisher's LDS multiple comparison test, and significant P values are shown in the graph.

The effect of the H_1_R antagonist on MAP2 was corroborated by immunohistochemistry and Western blot analysis in tissue samples obtained from the dorsal telencephalons of embryos from diabetic rats (Figure 8). As shown previously, the distributions of both neuron markers were altered in the diabetic group: MAP2 extended down from the marginal zone, and βIII-Tub exhibited a basal distribution corresponding to the marginal zone (Figures 8E,F). Diabetic rats treated with chlorpheniramine showed the characteristic cortical plate mark for MAP2 and βIII-Tub, but for βIII-Tub an inspected mark in the ventricular zone (Figures 8G,H). Furthermore, Western blot analysis demonstrated that chlorpheniramine partially prevented the increase in the MAP2 level in the diabetic group without affecting the protein level of βIII-Tub (Figures 8I–L).

## Discussion

To the best of our knowledge, embryonic glucose levels have not been previously reported in diabetic and control rat embryos. Here, we demonstrate that embryos from diabetic rats have significantly higher glucose levels than control embryos and that this phenomenon can be observed as early as E12. Nevertheless, embryo glycemia is lower than the glucose levels in pregnant rat serum at E12. The higher glucose levels observed in embryos from diabetic rats could be attributed to an increase in the expression of glucose transporter 3 (GLUT3) in the placentas of diabetic rats (Boileau et al., 1995).

The effects of high glucose concentrations on neuron differentiation are controversial. Some authors have reported increased differentiation in mice at E11.5 (both *in vitro* and *in vivo*) as a result of increased expression of the neurogenic factors Ngn1/2 and MAP2, decreased progenitor factors Hes1 and nestin, and changes in both the pattern and level of expression of Sonic Hedgehog and bone morphogenetic protein-4 (BMP4; Liao et al., 2004; Fu et al., 2006). In contrast, other groups have suggested that maternal diabetes promotes reduced neuron diferentiation related to the decreased expression of BMP4, nuclear factor (erythroid-derived 2)-like 2 (Nrf2), nuclear receptor binding SET domain protein 1 (Nsd1), paired box 3 (Pax3), hypoxia inducible factor 1 alpha subunit (Hif1a), cyclic adenosine monophosphate (cAMP) responsive element binding protein 1, and doublecortin (Liao et al., 2004; Pavlinkova et al., 2009; Salbaum and Kappen, 2010; Ejdesjo et al., 2011).

Discrepancies between these studies may be attributable to a variety of causes, including: timing during development, tissue origin within the neural tube, the presence (or absence) of NTDs in the embryos used, and erroneous inferences about mRNA levels and associated proteins, which depend on mRNA stability, protein half-life, and the action of translation regulators (de Sousa Abreu et al., 2009; Diaz et al., 2014). For example, we found that βIII-Tub was overexpressed in the diabetic group, even though we did not find any differences in the proteins associated with it.

Our findings support increased neuron differentiation in the dorsal telencephalon at the neurogenic peak (E14) in embryos from diabetic rats based on the increased levels of Ngn1, βIII-Tub, and MAP2 (determined via qRT-PCR) and the protein content (MAP2 via immunohistochemistry and Western blot analyses).

Although MAP2 is considered a mature neuron marker, high- and low-molecular weight isoforms are expressed differentially during the development of the CNS and adult tissue. Low-molecular weight isoforms (70 and 75 kDa, MAP2c/b, respectively) are highly expressed during embryo development and the early postnatal period (Garner et al., 1988; Riederer and Innocenti, 1992). In contrast, the two high-molecular weight isoforms are expressed throughout life (MAP2a) or predominantly in the adult brain (MAP2d; >270 kDa; Chung et al., 1996; Fujimori et al., 2002).

The presence of high- and increased low-molecular weight isoforms (as evidenced by the Western blots of diabetic rat embryo tissues) suggests that an early neuron maturation process may occur in addition to increased neuron differentiation. An alternative splicing process for this protein may also arise under our experimental conditions (Kalcheva and Shafit-Zagardo, 1995), as observed in fetal cardiac pathogenesis under diabetic conditions (Verma et al., 2013).

As previously reported, HA acts as a neurogenic factor in cortical NSC via H_1_R activation, promoting MAP2 and FOXP2 phenotypes. This action also increases neuron commitment by increasing the levels of Ngn1 and Prox1 during NSC proliferation (Molina-Hernandez and Velasco, 2008; Rodriguez-Martinez et al., 2012). Furthermore, *in utero* treatment with chlorpheniramine (H_1_R antagonist/inverse agonist) at E12 promotes reduced βIII-Tub and FOXP2 immunoreactivity in the dorsal telencephalon at E14 (Molina-Hernandez et al., 2013).

Our results suggest that increased levels of HA at E14 and/or H_1_R expression at E12 may be related to alterations in neuron differentiation in the diabetic model. The placenta may be the main source of the increased HA observed in embryos from diabetic dams. Alternatively, this increased HA level could be attributed to reduced fetal HA catabolism; however, no changes in histamine catabolism have been reported in tissues from STZ diabetic rats with increased HA concentrations (Gill et al., 1990).

Although HA is highly increased at E14 under hyperglycemia, the increased expression of H_1_R at E12 may be responsible for the increased neuron differentiation at E14, because the neurogenic effect of HA depends on the activation of this receptor (Molina-Hernandez and Velasco, 2008; Molina-Hernandez et al., 2013). This hypothesis was supported by the systemic administration of chlorpheniramine, which prevented the increased expression of MAP2 and βIII-Tub mRNA and MAP2 protein level in dorsal telencephalic tissue from diabetic rats. Although, previous reports regarding the participation of H_1_R on fetal and adult NSC in neuron differentiation are convincing (Yasuda and Yasuda, 1999; Molina-Hernandez and Velasco, 2008; Bernardino et al., 2012; Rodriguez-Martinez et al., 2012; Molina-Hernandez et al., 2013), we cannot reject a muscarinic participation using chlorpheniramine (Yasuda and Yasuda, 1999) since M2 receptor also enhances neural proliferation and neuron differentiation in E14 NSC *in vitro* (Williams et al., 2004; Zhou et al., 2004). Nevertheless, muscarinic binding sites appear between E13 and E14 in rats (Schlumpf et al., 1991) and their expression before this moment have not been reported. These data suggest that the effect reported here of chlorpheniramine may be indeed by H_1_R antagonism.

Low levels of HA and the effect of chlorpheniramine administration at E12 in the diabetic group suggest constitutive activity of H_1_R and inverse agonism of chlorpheniramine. Constitutive activity has been described for H_1_R in allergies (Nijmeijer et al., 2010) and over-expressing heterologous systems (Bakker et al., 2000, 2001), indicating that these phenomena are receptor density dependent.

In addition to the important role of H_1_R during CNS development, the possibility that the receptor exhibits constitutive activity in embryos from diabetic dams may have important consequences not only in the context of pregnancy and fetal CNS development but also regarding the development of other organs (i.e., the heart).

Here, we show changes in the fetal ontogeny of the histaminergic system in a maternal pathological condition that might be related with the increased neurogenesis in the dorsal telencephalon. The functional implication of an increased telencephalic neuron differentiation and/or neuron maturation is difficult to establish and, even more to relate it with the etiology of neurodevelopmental disorders, since several intrinsic, epigenetic, and environmental factors may influence neuronal development and potentially contribute to neurological and mental disorders. But it is possible that the increased telencephalic neurogenesis which is intimately related to cell proliferation and migration will have important consequences on laminar specification and neural circuit integration, aspects that will be further studied. Additionally, this is the first study revealing the expression of H_1_R and HDC as early as E12 and to demonstrate that HDC is active in the transitory fetal histaminergic system. This study opens up an important field of research regarding the participation of HA and H_1_R receptor in early corticogenesis in health and disease.

## Author contributions

All authors participated in the development of this research and drafting of the manuscript. KS and LM, performed the experiments and participated in the analysis of results. GG-L participate in immunohistochemistry experiments and analysis. WP animal acquisition and manipulation. ND, WP, and MDN-O, conception contributions, critical revision of the manuscript and experimental supervision. AM-H funding, experimental design, experimental and data supervision, data analysis and final approval of the manuscript.

### Conflict of interest statement

The authors declare that the research was conducted in the absence of any commercial or financial relationships that could be construed as a potential conflict of interest.

## Abbreviations

H_1_R: histamine H1 receptor
HA: histamine
MAP2: microtubule associated protein 2
FOXP2: forkhead box protein 2
βIII-Tub: BetaIII-tubulin
*Ngn*: neurogenin
NTDs: neural tube defects
NSC: neural stem cells
CNS: central nervous system
E: embryo day
Mash1: Achaete-Scute family basic helix-loop-helix (bHLH) transcription factor 1
Hes: Hes family bHLH transcription factor
Prox1: Prospero 1
GAPDH: glyceraldehyde 3-phosphate dehydrogenase
F: forward
R: reverse
HDC: histidine decarboxylase
GLUT3: glucose transporter 3
BMP4: bone morphogenetic protein 4
Nrf2: nuclear factor (erythroid-derived 2)-like 2
Nsd1: nuclear receptor binding SET domain protein 1
Pax3: paired box 3
Hif1a: hypoxia inducible factor 1 alpha subunit.
